# Eye movements predict treatment response in children and adolescents with social anxiety disorder

**DOI:** 10.1192/j.eurpsy.2025.1021

**Published:** 2025-08-26

**Authors:** J. L. Kleberg, M. Cervin, M. Nord, E. Serlachius, J. Högström

**Affiliations:** 1 Department of Psychology, Stockholm University, Stockholm; 2 Department of Clinical Sciences, Lund University, Lund; 3 Department of Public Global Health; 4 Department of Clinical Neuroscience, Karolinska Institutet, Stockholm, Sweden

## Abstract

**Introduction:**

Response to treatment in pediatric social anxiety disorder (SAD) is highly variable. Atypical face processing has been suggested as a maintaining factor. A previous study reported that youth with SAD scan a more restricted area of faces than healthy controls during emotion recognition, potentially interfering with social cognition.

**Objectives:**

The current study examined whether visual scanning and arousal (pupil dilation) also predicts treatment response and changes as a function of successful treatment.

**Methods:**

Youth with SAD (n = 55) were assessed prior to treatment with internet-delivered cognitive behavioral therapy (ICBT) or supportive therapy (ISUPPORT) and three and twelve months after treatment.

**Results:**

Restricted scanning of faces predicted worse treatment outcome, most consistently for youth receiving ICBT. No evidence for a change in social attention after treatment was found. Instead, visual social attention measures showed moderate to high stability. Children whose arousal decreased from baseline to three months follow up were most likely to benefit from ICBT rather than ISUPPORT, pointing to a role of arousal in treatment response.

**Conclusions:**

Restricted scanning of faces may interfere with social interaction, thereby interfering with treatment. These results have implications for our understanding of social information processing in SAD.

**Disclosure of Interest:**

None Declared

